# The way of Qu‐making significantly affected the volatile flavor compounds in Huangjiu (Chinese rice wine) during different brewing stages

**DOI:** 10.1002/fsn3.2835

**Published:** 2022-04-07

**Authors:** Qi Peng, Huajun Zheng, Kai Meng, Yimeng Zhu, Wenxia Zhu, Hongyi Zhu, Chi Shen, Jianwei Fu, Nabil l. Elsheery, Guangfa Xie, Jiongping Han, Peng Wu, Yuyan Fan, DulaBealu Girma, Jianqiu Sun, Baowei Hu

**Affiliations:** ^1^ 66326 National Engineering Research Center for Chinese CRW (branch center) Shaoxing University Shaoxing China; ^2^ California Institute of Food and Agricultural Research University of California Davis California USA; ^3^ 68781 Agricultural Botany Department Faculty of Agriculture Tanta University Tanta Egypt; ^4^ College of Biology and Environmental Engineering College of Shaoxing CRW Zhejiang Shuren University Hangzhou China; ^5^ 66326 School of Medicine Shaoxing University Shaoxing China; ^6^ School of Environmental Science and Engineering Suzhou University of Science and Technology Suzhou China

**Keywords:** brewing stage, HS–SPME–GC–MS, Huangjiu, volatile flavor compounds, wheat Qu

## Abstract

The volatile flavor compounds of Huangjiu (Chinese rice wine) brewed from different raw materials were obviously different, but there were few studies on the volatile flavor compounds of Huangjiu brewed from different wheat Qu at different brewing stages. In this paper, headspace–solid phase microextraction combined with gas chromatography–mass spectrometry, combined with principal component analysis and sensory evaluation, was used to determine the volatile flavor compounds in Huangjiu brewed from wheat Qu made by hand and wheat Qu made by mechanical. The results showed that there were significant differences in the contents and types of volatile flavor substances in Huangjiu brewed from different wheat Qu at fermentation stages, and the prefermentation and postfermentation Huangjiu samples could be well distinguished from each other. Compared with the Huangjiu brewed from wheat Qu made by mechanical, the Huangjiu brewed from wheat Qu made by hand has stronger aroma and better taste.

## Graphical abstract

In this paper, headspace–solid phase microextraction combined with gas chromatography–mass spectrometry (HS–SPME–GC–MS), combined with principal component analysis (PCA) and sensory evaluation, was used to determine the volatile flavor compounds in Huangjiu (Chinese rice wine ) brewed from wheat Qu made by hand and wheat Qu made by mechanical. The results showed that compared with the Huangjiu brewed from wheat Qu made by mechanical, the Huangjiu brewed from wheat Qu made by hand has stronger aroma and better taste.

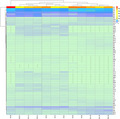

## 1 INTRODUCTION

Huangjiu (Chinese rice wine) is made from grain as the main raw material, wheat Qu or xiao Qu as saccharifying and fermenting agent (Ouyang et al., 2016), and is brewed from the processes of soaking rice, steamed rice, prefermentation, and postfermentation (Jiao et al., 2017). Huangjiu, with its unique flavor and high nutritional value (Ren et al., 2019; Zhao et al., 2018), is the national wine of modern China, and is known as one of the three ancient wines in the world. As a traditional Chinese alcoholic beverage (Gong et al., 2019; Yu et al., 2012), there are many varieties of Huangjiu, but due to the differences in various aspects such as wheat Qu and brewing technology, the final style of Huangjiu of different varieties is also different.

“Making wheat Qu with wheat and brewing wine with wheat Qu” is a traditional operation technology of Huangjiu brewing technology (GB/T13662‐2018, 2018), the quality of wheat Qu can directly affect the quality and yield of Huangjiu (Shen, Yang, et al., 2012; Xu et al., 2010). Wheat Qu contains abundant microorganisms and enzymes (Zhang et al., 2019), such as fungi, yeast, bacteria, saccharifying enzymes, and proteases (Plata et al., 2005), and these microorganisms play a very important role in the flavor of Huangjiu. Nowadays, wheat Qu is divided into many types. According to different ways of making wheat Qu, wheat Qu can be divided into wheat Qu made by hand and wheat Qu made by mechanical. There are more or less differences in the flavor of Huangjiu brewed from these two kinds of wheat Qu. Thus, it is of great significance to study the volatile flavor compounds of Huangjiu brewed from different wheat Qu to control the flavor of Huangjiu in the industrial production process. Thus, it is of great significance to study the volatile flavor compounds of Huangjiu brewed from different wheat Qu to control the flavor of Huangjiu in the industrial production process.

With the development of economic globalization, Huangjiu has spread widely throughout the international market. Huangjiu is a complex mixture composed of hundreds of flavor compounds. The aroma of Huangjiu is one of the decisive factors affecting consumers’ sensory quality and purchase (Shen, Zhu, et al., 2021; Shen, Wu, et al., 2021; Welke et al., 2014). The aroma components of Huangjiu wine mainly come from raw materials, substances decomposed by various enzymes, and microbial metabolites in the brewing process (Chen & Xu, 2013; Shen, Li, et al., 2012). At present, although there are many studies on the volatile flavor compounds of Huangjiu brewed with different raw materials, qualities, or technologies (Chen & Xu, 2012; Yong et al., 2013). However, there are few studies and experiments on the variation of volatile flavor compounds in different brewing stages of Huangjiu brewed from wheat Qu made by hand and wheat Qu made by mechanical. Therefore, it is necessary to study the changes of volatile flavor compounds in Huangjiu brewed from different wheat Qu during the different brewing stages.

In this study, HS–SPME–GC–MS (headspace–solid‐phase microextraction combined with gas chromatography–mass spectrometry) was used to compare the composition changes of volatile flavor substances in Huangjiu brewed by wheat Qu made by hand and wheat Qu made by mechanical (Wang et al., 2014). The Huangjiu was brewed with the wheat Qu made by hand provided by Zhejiang Guyue Longshan Shaoxing Wine Co., Ltd. and the wheat Qu made by mechanical provided by Kuaijishan Shaoxing Wine Co., Ltd. The concentrations of ethanol, total acidity, and reducing sugar were determined. I hope this research can help provide more accurate critical control points for the production of better‐flavored Huangjiu while preserving the flavor and superior quality characteristics of traditional Huangjiu, which will also have significance for the monitoring of Huangjiu production.

## 2 MATERIAL AND METHODS

### 2.1 Wheat Qu and chemicals

The Wheat Qu made by hand provided by Shaoxing Guyue Longshan Co., Ltd. and the Wheat Qu made by mechanical method provided by Kuaijishan Shaoxing Wine Co., Ltd. were used for brewing Huangjiu. All reagents and standards were purchased at Sigma‐Aldrich (Shanghai, China).

### 2.2 Preparation of Wheat Qu

**FIGURE 1:**
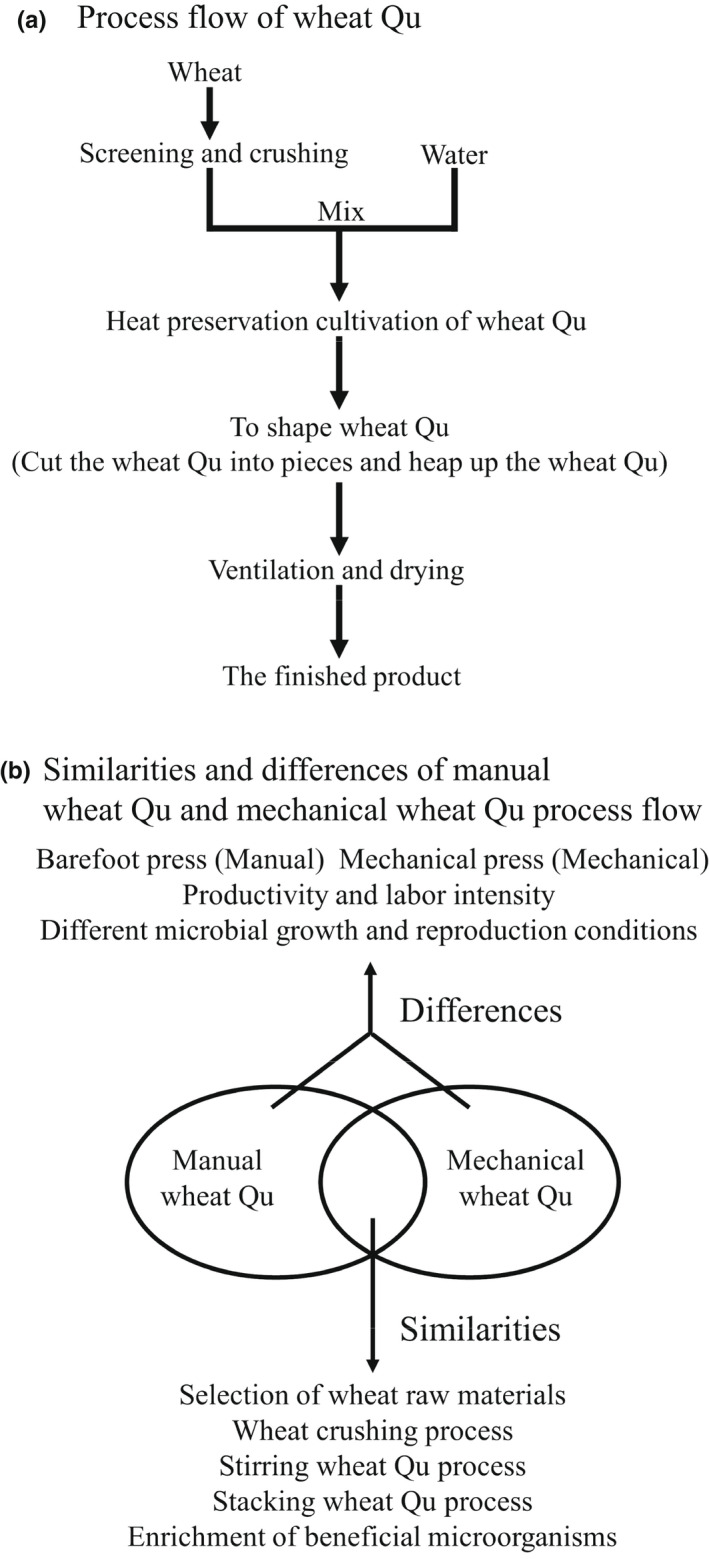
Technological process of wheat Qu (a,b). (a) Process flow of wheat Qu. (b) Similarities and differences of manual wheat Qu and mechanical wheat Qu process flow

Figure 1(a,b). The mechanical Wheat Qu process is basically the same as the manual Wheat Qu process, but the biggest difference is that the mechanical Wheat Qu process uses mechanical devices to replace part of the operating procedures in the manual Wheat Qu process (Zhang et al., 2019).

### 2.3 Huangjiu making

There are two groups of Huangjiu samples in the experiment. One group is Huangjiu brewed from wheat Qu made by hand and the other group is Huangjiu brewed from wheat Qu made by mechanical method.

### 2.4 Sample collection

In the process of fermentation, according to different times, samples were collected separately. The sampling time of Huangjiu brewed by handmade wheat Qu was 2, 4, 6 days of the primary fermentation and 10, 20, 30 days of the postfermentation (Because of the long fermentation time and short sampling interval of the Huangjiu made by hand, it cannot reflect the change of flavor components). The sampling time of Huangjiu brewed by mechanical method wheat Qu was 2, 4, 6 days of the primary fermentation and 10, 14, 18 days of the postfermentation. (Because of the short fermentation time and long sampling interval of the Huangjiu made by mechanical methods, it cannot reflect the change of flavor components). The fermented mash was filtered and centrifuged, and then the chemical and flavor indicators of the supernatant were analyzed.

### 2.5 Determination of chemical indices

The content of reducing sugar in rice wine samples was determined by the dinitrosalicylic acid method (Miller, 1959). Add 10 ml of Huangjiu sample into 50 ml of deionized water and determine the total acidity (in lactic acid) with 0 titration method 0.1 mol/L sodium hydroxide, until the end pH reaches 8.2 (GB/T13662‐2018, 2018). Determination of ethanol content in Huangjiu is carried out by the distillation method (GB/T13662‐2018, 2018).

### 2.6 Volatile compound analysis

The SPME units and fibers (50 µm divinylbenzene (DVB)/carboxen (CAR)/polydimethylsiloxane (PDMS)) are from Supelco (Bellefonte, PA, USA). Each Huangjiu sample was diluted with deionized water to a final ethanol concentration of 6% (v/v) for accurate GC–MS analysis. In different stages, 2 g of sodium chloride was added into 5ml of sample and put into 20 ml headspace glass vials with screw caps, add 5 ml of internal standard (2‐octanol, mass concentration 89.65 μg/L). The fibers were introduced into the SPME device, inserted into a headspace glass bottle, and shaken at 50°C for 30 min to extract and absorb volatile compounds. Then they were desorbed within 7 min at 250°C into the GC inlet with the automatic autosampler. A gas chromatograph (GC) with an attached mass spectrometer (SCION SQ‐456; Bruker Daltonics Inc., USA) was used with a DB‐Wax column (60 m*0.25 mm*0.25 μm; Agilent Technologies, USA). As the carrier gas, helium was delivered at a flow rate of 1 ml/min. The gas chromatograph oven temperature was initially maintained at 40°C for 3 min, increased to 120°C at a rate of 6°C/min and then increased to 230°C at a rate of 8°C/min, and finally held for 15 min. The mass detector was operated in electron impact mode at an ionizing voltage of 70 eV. The ion source temperature was set at 220°C (Jung et al., 2014). The volatile components were identified by mass spectrometry (MS) and quantified by internal standard (2,4,6‐trimethylpyridine, 1 mg/L). The relative content of each component was counted according to the area normalization method. The results were reported in the mean value of three replicates of Huangjiu samples (Yang et al., 2017).

### 2.7 DNA extraction and sequencing

DNA extraction and sequencing were performed according to Xie, GF method (Xie et al., 2021). A linear relationship was established between the sequencing data and flavor substances by partial least squares regression. The X variable is relative abundance of bacterial community. The Y variable is the average concentration of flavor substances (Shen, Zhu, et al., 2021; Shen, Wu, et al., 2021).

### 2.8 Sensory analysis

Sensory evaluation of the Huangjiu was conducted by a panel of 10 judges (5 males and 5 females) with rich experience in wine descriptive analysis. There was a uniform source of lightening, absence of noise, and distracting stimuli. Fifteen milliliter samples were presented in clear, tulip‐shaped glasses marked with a random order and covered with petri dishes. A total of 11 descriptors of appearance (color and turbidity), aroma (alcohol, fruit, and cereal), taste/flavor (sweet, sour, and bitter), and mouthfeel (astringency, continuation, and full body) were selected by the panel during preliminary training sessions to describe the samples. Standards used to define the aromas and taste descriptor were included in the training and wine‐tasting test. Intensity ratings were scored on a scale from 0 to 9 (0: none; 1–2: very weak; 3–4: ordinary; 5–6: moderate; 7–8: strong; 9: very strong).

### 2.9 Statistical analysis

The chemical composition and flavor of each rice wine were analyzed, and the analysis was repeated three times, and the results were expressed as the average value ± standard deviation. SPSS ver. 19.0 (SPSS Inc.), Origin 2019b (Origin Lab Inc.), and Microsoft Office Excel 2007 (Microsoft Corp.) were used to process the GC–MS and chemical indicator data. PCA using a correlation matrix with no rotation was performed to investigate the relationships between the different brewing stages of Huangjiu and the volatile flavor compounds.

## 3 RESULTS AND DISCUSSION

### 3.1 Analysis of general chemical indicators during fermentation

The contents of ethanol, total acidity, and reducing sugar in Huangjiu samples are shown in Table 1. The content of ethanol and reducing sugar in Huangjiu can reflect the liquefaction and saccharification of wheat Qu, thus reflecting the fermentation performance of wheat Qu. Alcohol is the carrier of aroma and flavor substances in Huangjiu, which can make Huangjiu have the mellow aroma. The experimental results showed that the alcohol content of Huangjiu brewed from wheat Qu made by hand was 15.61% vol, which was significantly lower than that of Huangjiu brewed from wheat Qu made by mechanical was 17.13% vol; correspondingly, the reducing sugar content of Huangjiu brewed from wheat Qu made by hand was 4.01 g/L, which was significantly higher than that of Huangjiu brewed from wheat Qu made by mechanical which was 1.13 g/L. This is because in the process of Huangjiu fermentation, the reducing sugar in the mash is converted to ethanol through the glycolytic pathway (Embden–Meyerhof–Parnas (EMP)) by the action of yeast (Zhang, Wang, et al., 2019). This indicates that the liquefaction and saccharification forces of wheat Qu made by mechanical are obviously stronger than those of wheat Qu made by hand.

**TABLE 1:** Results of chemical analysis in Huangjiu brewed by different wheat Qu

Chemical index	Wheat Qu made by hand	Wheat Qu made by mechanical method
Ethanol (% v/v)	15.61 ± 0.49	17.13 ± 0.50
Acidity (as lactic acid, g/L)	3.16 ± 0.05	3.53 ± 0.02
Reducing sugar (g/L)	4.01 ± 0.11	1.13 ± 0.08

Note

The results are expressed as mean ± standard deviation, and each sample was repeated three times.

Acidity is an important index to judge the fermentation degree of Huangjiu. “No acid, no taste” indicates that acidities are also an important part of flavor compounds in Huangjiu. Proper amount of acid can not only enhance the taste of Huangjiu, but can also make the color and aroma of Huangjiu more harmonious and plump (Yu et al., 2012). Therefore, acidity detection is an essential management method during Huangjiu fermentation. By analyzing the acid content of Huangjiu, it is helpful to know the fermentation situation of Huangjiu in time and ensure the normal fermentation process. Table 1 shows that the acidity of Huangjiu brewed from wheat Qu made by hand was 3.16 g/L, which is lower than the acidity of the Huangjiu brewed from wheat Qu made by mechanical which was 3.53 g/L. Compared with the Huangjiu brewed from wheat Qu made by hand, the Huangjiu brewed from wheat Qu made by mechanical had higher total acidity and lower residual sugar, which indicated that the Huangjiu brewed from wheat Qu made by mechanical had excellent fermentation performance. In our previous study, we found that the abundance of lactic acid bacteria in the two kinds of wine is different due to the different microorganisms brought in by different wheat Qu, which is one of the main reasons for the inconsistent acidity of rice wine.

### 3.2 Volatile flavor compounds in the different fermentation stages of Huangjiu making

Qualitative analysis of the main aroma compounds in the Huangjiu made from wheat Qu by hand and in the Huangjiu made from wheat Qu by mechanical method during manufacture is presented in Tables 2 and 3. Sixty major flavor compounds were identified, which included 18 alcohols, 26 esters, 9 aldehydes, and 7 acids.

**TABLE 2:** The contents of volatile flavor compounds during different brewing stages of Huangjiu fermented with the Huangjiu made from wheat Qu by hand which are analyzed by headspace–solid phase microextraction combined with gas chromatography–mass spectrometry (HS–SPME–GC–MS)

	Volatile matter		2 days of the primary fermentation	4 days of the primary fermentation	6 days of the primary fermentation	10th day of the postfermentation	20th day of the postfermentation	30th day of the postfermentation
Alcohols	A1	Ethanol	260.52 ± 31.82	292.48 ± 42.72	265.75 ± 46.87	274.69 ± 51.19	259.74 ± 38.91	281.94 ± 41.23
	A2	Isobutanol	14.37 ± 2.67	15.23 ± 3.13	17.34 ± 4.25	15.71 ± 2.43	14.12 ± 3.63	15.33 ± 3.91
	A3	Isopentanol	53.72 ± 11.23	52.65 ± 17.59	47.23 ± 16.41	45.46 ± 12.92	48.24 ± 13.26	44.18 ± 14.23
	A4	β‐phenylethanol	50.78 ± 9.57	51.60 ± 11.23	45.73 ± 12.81	42.25 ± 11.62	38.51 ± 8.43	48.17 ± 12.86
	A5	2,3‐Butanediol	3.15 ± 1.11	4.22 ± 1.63	5.07 ± 1.08	3.45 ± 2.09	2.56 ± 1.23	3.88 ± 0.96
	A7	N‐octanol	6.36 ± 2.43	8.30 ± 1.91	5.16 ± 1.54	8.18 ± 1.52	7.27 ± 2.71	8.53 ± 2.09
	A8	Nonyl alcohol	4.08 ± 2.23	5.67 ± 1.41	8.87 ± 1.51	7.73 ± 1.89	4.51 ± 1.75	7.13 ± 3.23
	A9	Furfuryl alcohol	5.23 ± 1.06	4.23 ± 0.95	5.14 ± 2.85	6.46 ± 1.54	5.21 ± 1.91	6.12 ± 2.41
	A10	Propanol	4.28 ± 0.88	3.70 ± 1.01	4.53 ± 1.27	3.88 ± 2.93	4.82 ± 2.55	5.76 ± 1.41
	A11	2‐Methyl‐1‐propanol	2.53 ± 2.02	3.15 ± 1.86	2.62 ± 1.08	4.98 ± 2.57	7.21 ± 2.15	6.18 ± 3.51
	A12	Butanol	3.14 ± 1.34	4.32 ± 1.2	6.25 ± 1.01	5.16 ± 1.83	4.66 ± 3.09	7.36 ± 4.01
	A13	Hexanol	3.87 ± 1.83	5.23 ± 2.07	4.23 ± 2.14	6.53 ± 2.62	5.77 ± 2.01	5.56 ± 1.98
	A14	Octanol	2.15 ± 1.23	2.51 ± 2.62	7.75 ± 1.83	6.88 ± 1.51	3.44 ± 1.82	1.62 ± 1.24
	A15	Benzyl alcohol	2.95 ± 0.98	4.52 ± 1.51	4.87 ± 1.51	5.67 ± 1.62	7.28 ± 1.19	6.36 ± 2.53
	A16	Heptadiene glycol	3.63 ± 2.34	3.87 ± 2.57	3.60 ± 1.01	3.60 ± 2.89	4.84 ± 3.01	3.60 ± 3.12
	A17	1‐Octene‐3‐ol	2.53 ± 1.14	4.63 ± 1.25	3.80 ± 1.61	4.21 ± 1.67	4.37 ± 1.56	5.11 ± 0.99
	A18	D‐citronellol	3.59 ± 2.11	8.82 ± 2.27	9.36 ± 2.89	7.44 ± 1.11	6.46 ± 2.01	7.75 ± 3.91
	A20	3‐Methylthiopropanol	3.71 ± 2.81	3.22 ± 2.39	4.81 ± 2.11	4.68 ± 3.20	4.84 ± 3.15	4.39 ± 1.67
	Total alcohols		430.16 ± 78.43	478.31 ± 101.04	452.09 ± 106.66	456.94 ± 108.45	433.80 ± 96.53	468.92 ± 111.23
Esters	B1	Ethyl acetate	22.95 ± 11.23	26.24 ± 13.14	27.06 ± 12.47	28.66 ± 10.26	27.52 ± 12.45	28.85 ± 13.44
	B2	Ethyl lactate	12.73 ± 9.41	13.13 ± 11.18	16.41 ± 13.75	26.84 ± 12.61	30.34 ± 11.23	32.50 ± 16.49
	B3	Ethyl butyrate	17.24 ± 7.79	16.35 ± 5.15	18.72 ± 9.22	22.95 ± 11.56	25.92 ± 9.91	30.61 ± 13.16
	B4	Ethyl propionate	1.72 ± 0.81	1.61 ± 0.55	1.94 ± 0.41	2.07 ± 0.92	1.83 ± 1.67	1.41 ± 0.56
	B5	Ethyl valerate	0.65 ± 0.29	0.91 ± 0.41	1.15 ± 0.27	1.25 ± 0.91	1.82 ± 0.87	2.01 ± 0.78
	B6	Propyl hexanoate	1.01 ± 0.13	1.04 ± 0.31	0.92 ± 0.50	1.11 ± 0.56	1.15 ± 0.65	1.45 ± 0.82
	B7	Ethyl heptanoate	0.91 ± 0.66	0.80 ± 0.77	1.23 ± 0.87	1.08 ± 0.90	1.10 ± 0.54	1.25 ± 0.72
	B8	Hexyl acetate	3.12 ± 1.18	2.95 ± 1.23	2.98 ± 1.03	4.02 ± 1.06	3.83 ± 0.91	4.08 ± 1.15
	B9	Hexyl caproate	0.45 ± 0.21	0.27 ± 0.41	0.65 ± 0.49	0.97 ± 0.38	1.01 ± 0.46	1.14 ± 0.81
	B10	Gamma nonyllactone	0.51 ± 0.29	0.82 ± 0.66	1.28 ± 0.71	0.95 ± 0.26	1.11 ± 0.63	1.28 ± 0.48
	B11	Isopentyl hexanoate	0.94 ± 0.51	0.87 ± 0.14	1.27 ± 0.65	2.66 ± 0.81	1.90 ± 0.89	2.06 ± 0.77
	B12	Ethylene caproate	1.99 ± 1.01	2.07 ± 1.99	3.07 ± 1.93	3.74 ± 1.87	3.88 ± 1.73	4.13 ± 1.98
	B13	Isoamyl lactate	0.92 ± 0.81	1.92 ± 0.72	0.91 ± 0.67	1.72 ± 0.78	1.97 ± 0.98	2.02 ± 1.08
	B14	Monoethyl succinate	1.09 ± 0.91	1.24 ± 1.38	1.76 ± 1.15	1.96 ± 1.81	2.07 ± 0.50	2.64 ± 1.11
	B15	Ethyl benzoate	3.03 ± 1.23	3.79 ± 0.24	3.98 ± 1.23	4.42 ± 1.48	5.06 ± 0.70	5.84 ± 1.61
	B16	Phenethyl formate	3.07 ± 1.03	3.08 ± 0.81	2.99 ± 0.44	4.59 ± 0.77	4.08 ± 0.65	4.50 ± 1.18
	B17	Ethyl phenylacetate	5.10 ± 2.05	3.06 ± 1.47	4.21 ± 0.94	4.07 ± 1.99	5.13 ± 1.93	6.06 ± 1.83
	B18	Phenylethyl acetate	3.06 ± 1.10	2.60 ± 0.78	2.09 ± 1.22	3.74 ± 1.07	4.02 ± 0.66	4.13 ± 1.54
	B19	Ethyl cetane	2.14 ± 1.07	1.53 ± 1.22	1.76 ± 1.27	2.24 ± 1.85	2.08 ± 1.56	2.07 ± 1.47
	B20	Octadecarbonate	1.01 ± 0.87	1.09 ± 0.73	1.19 ± 1.02	1.50 ± 1.18	1.86 ± 1.31	1.65 ± 1.27
	B21	Diethyl succinate	2.03 ± 0.88	3.08 ± 1.04	3.22 ± 1.27	2.96 ± 1.65	2.65 ± 2.07	3.13 ± 1.76
	B22	Isoamyl acetate	5.24 ± 2.56	6.75 ± 1.99	6.84 ± 3.05	7.22 ± 2.97	6.11 ± 3.14	7.19 ± 2.04
	B23	Ethyl myristate	7.74 ± 3.09	6.09 ± 3.07	8.36 ± 3.83	7.19 ± 3.65	8.03 ± 3.07	5.21 ± 2.17
	B24	Acetic acid‐2‐phenylethyl ester	2.23 ± 0.99	3.13 ± 1.28	2.64 ± 1.81	3.06 ± 1.34	2.05 ± 1.87	2.15 ± 1.65
	B25	Ethyl decanoate	2.98 ± 1.01	3.22 ± 1.22	4.03 ± 1.65	3.84 ± 1.49	3.87 ± 2.03	3.96 ± 1.58
	B26	Ethyl undecanoate	1.08 ± 0.39	1.10 ± 0.88	1.76 ± 0.93	2.07 ± 1.05	1.56 ± 1.05	2.14 ± 1.67
	Total esters		104.93 ± 51.89	108.75 ± 52.98	122.41 ± 63.10	146.88 ± 65.39	151.96 ± 62.76	163.44 ± 73.46
Aldehyde	C1	Benzaldehyde	3.47 ± 1.24	3.84 ± 1.27	3.12 ± 1.05	2.84 ± 1.11	2.52 ± 1.83	2.39 ± 1.47
	C2	Phenylacetaldehyde	2.34 ± 0.97	2.65 ± 1.13	3.02 ± 2.01	2.92 ± 1.53	2.09 ± 1.07	2.51 ± 1.25
	C3	Furfural	3.81 ± 2.09	3.73 ± 1.07	3.58 ± 1.99	2.95 ± 1.37	2.62 ± 1.64	2.39 ± 0.41
	C4	Nonanal	2.24 ± 1.07	1.72 ± 0.63	1.25 ± 0.66	0.88 ± 0.43	0.25 ± 0.26	0.46 ± 0.19
	C5	Decanal	1.13 ± 0.83	1.25 ± 0.86	1.07 ± 0.24	1.26 ± 0.80	0.56 ± 0.30	0.70 ± 0.40
	C6	2‐Phenyl‐2‐butyraldehyde	1.03 ± 0.26	1.86 ± 1.08	0.63 ± 0.29	ND	ND	ND
	C7	5‐Methylfurfural	1.17 ± 0.77	1.03 ± 0.62	0.83 ± 0.14	ND	ND	ND
	C8	3‐Methylbutyraldehyde	2.04 ± 1.03	2.54 ± 1.26	2.16 ± 1.02	1.07 ± 0.12	1.45 ± 1.28	1.27 ± 0.98
	C9	P‐Methylbenzaldehyde	2.98 ± 1.87	2.59 ± 1.08	2.35 ± 1.17	1.92 ± 0.87	1.63 ± 1.05	1.86 ± 0.63
	Total aldehydes		20.21 ± 10.15	21.23 ± 9.09	18.01 ± 8.76	13.84 ± 6.28	11.13 ± 7.50	11.57 ± 5.43
Volatile acids	D1	Acetic acid	11.61 ± 4.19	12.71 ± 4.85	9.03 ± 3.76	8.57 ± 4.16	6.64 ± 3.55	6.91 ± 3.47
	D2	Hexanoic acid	2.16 ± 1.07	3.06 ± 1.27	2.09 ± 1.09	1.08 ± 0.89	1.82 ± 0.67	1.35 ± 0.65
	D3	Valeric acid	9.25 ± 3.66	7.21 ± 3.01	6.61 ± 3.15	5.24 ± 2.14	4.02 ± 2.07	4.83 ± 1.97
	D4	Caprylic acid	0.86 ± 0.24	0.77 ± 0.41	0.88 ± 0.41	0.91 ± 2.20	0.73 ± 0.53	0.76 ± 0.34
	D5	Butyrate	1.23 ± 1.01	0.96 ± 0.87	0.18 ± 0.13	0.73 ± 0.35	0.95 ± 0.44	0.81 ± 0.31
	D6	Decanoic acid	2.07 ± 0.93	1.87 ± 1.13	1.12 ± 0.65	2.05 ± 1.01	1.83 ± 0.62	1.18 ± 0.86
	D7	3‐Methylbutanoic acid	4.76 ± 1.65	4.14 ± 1.63	3.84 ± 0.97	4.15 ± 2.07	2.83 ± 1.14	2.67 ± 1.31
	Total acid compounds		32.00 ± 12.81	30.72 ± 13.24	23.75 ± 10.27	22.73 ± 10.91	18.83 ± 9.09	18.51 ± 9.01
Total volatile flavor compounds			587.7 ± 149.13	639 ± 176.47	616.26 ± 188.87	640.39 ± 191.21	615.72 ± 176.97	6762.42 ± 194.39

Note

The results are expressed as means ± standard deviation with three replicates for Huangjiu sample during different brewing stages.

Abbreviation: ND, not detected.

**TABLE 3:** The contents of volatile flavor compounds during different brewing stages of Huangjiu fermented with the Huangjiu made from wheat Qu by mechanical method which were analyzed by headspace–solid phase microextraction combined with gas chromatography–mass spectrometry (HS–SPME–GC–MS)

	Volatile matter		2 days of the primary fermentation	4 days of the primary fermentation	6 days of the primary fermentation	10th day of the postfermentation	14th day of the postfermentation	18th day of the postfermentation
Alcohols	A1	Ethanol	303.16 ± 40.82	344.68 ± 31.70	299.61 ± 32.48	283.38 ± 31.99	270.13 ± 34.39	285.33 ± 26.87
	A2	Isobutanol	9.41 ± 5.38	10.61 ± 4.65	9.70 ± 2.86	10.13 ± 1.41	13.03 ± 3.02	14.12 ± 2.13
	A3	Isopentanol	73.60 ± 12.19	64.92 ± 13.38	63.20 ± 11.29	63.54 ± 10.46	61.82 ± 11.23	57.67 ± 12.54
	A4	β phenylethanol	64.77 ± 12.66	60.75 ± 10.57	64.97 ± 11.47	67.95 ± 15.27	56.67 ± 11.63	54.76 ± 12.75
	A5	2,3‐Butanediol	2.24 ± 0.41	2.71 ± 0.09	5.03 ± 2.83	3.13 ± 1.47	3.84 ± 1.19	3.11 ± 2.13
	A7	*N*‐octanol	3.64 ± 0.41	3.13 ± 1.66	2.93 ± 0.47	2.70 ± 1.18	1.75 ± 0.23	2.53 ± 1.19
	A8	Nonyl alcohol	3.33 ± 0.51	3.11 ± 0.25	2.51 ± 0.11	1.82 ± 0.06	1.09 ± 0.02	1.03 ± 0.41
	A9	Furfuryl alcohol	7.78 ± 0.67	8.43 ± 3.55	9.76 ± 3.84	10.36 ± 3.05	11.94 ± 6.61	15.06 ± 2.19
	A10	Propanol	5.96 ± 1.09	6.30 ± 1.22	5.46 ± 1.12	5.03 ± 1.62	4.41 ± 1.22	4.59 ± 1.43
	A11	2‐Methyl−1‐Propanol	1.56 ± 0.02	1.25 ± 0.17	1.33 ± 0.28	0.85 ± 0.08	0.61 ± 0.11	1.24 ± 0.42
	A12	Butanol	3.15 ± 0.05	2.83 ± 0.41	2.06 ± 0.13	1.84 ± 0.51	1.38 ± 0.23	2.24 ± 0.51
	A13	Hexanol	4.12 ± 0.27	3.52 ± 1.16	3.15 ± 0.32	2.51 ± 0.26	1.94 ± 0.19	1.83 ± 1.19
	A14	Octanol	1.84 ± 0.42	1.63 ± 0.11	1.42 ± 0.06	1.07 ± 0.24	0.85 ± 0.16	0.75 ± 0.06
	A15	Benzyl alcohol	1.54 ± 0.23	1.34 ± 0.02	1.03 ± 0.18	0.55 ± 0.28	0.67 ± 0.14	0.53 ± 0.25
	A16	Heptadiene glycol	4.81 ± 0.15	4.08 ± 1.44	3.78 ± 0.09	3.23 ± 1.25	2.81 ± 1.15	2.55 ± 1.24
	A17	1‐Octene−3‐ol	ND	ND	ND	ND	ND	ND
	A18	D‐citronellol	ND	ND	ND	ND	ND	ND
	A20	3‐Methylthiopropanol	ND	ND	ND	ND	ND	ND
	Total alcohols		490.87 ± 74.66	519.26 ± 70.91	475.90 ± 67.94	458.06 ± 70.66	432.90 ± 72.91	447.31 ± 70.91
Esters	B1	Ethyl acetate	25.42 ± 10.09	30.95 ± 10.26	32.65 ± 10.57	36.49 ± 10.36	37.38 ± 11.59	37.16 ± 11.19
	B2	Ethyl lactate	11.23 ± 5.57	16.44 ± 11.62	18.44 ± 6.09	23.88 ± 13.06	24.31 ± 12.53	22.28 ± 7.13
	B3	Ethyl butyrate	18.17 ± 7.84	22.06 ± 9.21	18.18 ± 8.26	21.01 ± 6.14	24.87 ± 9.27	25.85 ± 12.46
	B4	Ethyl propionate	1.17 ± 1.09	4.19 ± 1.53	5.05 ± 1.95	5.19 ± 1.87	6.74 ± 1.22	5.48 ± 1.16
	B5	Ethyl valerate	1.31 ± 0.44	1.84 ± 0.73	1.86 ± 0.11	3.52 ± 0.59	3.04 ± 0.45	3.83 ± 1.19
	B6	Propyl hexanoate	0.77 ± 0.03	0.83 ± 0.06	1.23 ± 0.03	1.84 ± 0.21	2.40 ± 0.86	2.48 ± 0.69
	B7	Ethyl heptanoate	0.61 ± 0.08	0.95 ± 0.18	1.80 ± 0.05	2.09 ± 0.45	3.82 ± 1.25	3.56 ± 1.31
	B8	Hexyl acetate	2.82 ± 1.05	3.08 ± 0.54	3.45 ± 10.23	4.22 ± 1.83	5.13 ± 0.64	4.78 ± 1.53
	B9	Hexyl caproate	0.26 ± 0.01	0.83 ± 0.02	1.11 ± 0.07	1.56 ± 0.13	1.94 ± 0.28	1.84 ± 0.42
	B10	Gamma nonyllactone	0.50 ± 0.13	0.79 ± 0.03	1.12 ± 0.44	1.59 ± 0.21	0.86 ± 0.01	1.04 ± 0.25
	B11	Isopentyl hexanoate	0.46 ± 0.15	0.85 ± 0.27	1.63 ± 0.41	1.84 ± 0.85	2.06 ± 0.23	2.51 ± 0.85
	B12	Ethylene caproate	1.85 ± 0.55	2.44 ± 0.21	2.81 ± 0.34	3.10 ± 1.85	3.82 ± 1.11	3.13 ± 1.12
	B13	Isoamyl lactate	0.82 ± 0.04	1.25 ± 0.67	1.47 ± 0.51	3.01 ± 2.85	2.80 ± 0.21	2.14 ± 1.63
	B14	Monoethyl succinate	0.84 ± 0.11	1.27 ± 0.33	2.03 ± 0.14	2.81 ± 0.4	2.99 ± 2.03	2.54 ± 0.85
	B15	Ethyl benzoate	2.83 ± 0.56	5.23 ± 0.54	6.17 ± 0.99	9.02 ± 0.43	9.72 ± 1.54	9.17 ± 2.63
	B16	Phenethyl formate	2.83 ± 0.24	4.08 ± 3.83	6.05 ± 3.41	6.51 ± 4.45	6.97 ± 2.36	6.36 ± 3.21
	B17	Ethyl phenylacetate	5.15 ± 2.16	7.36 ± 1.01	8.13 ± 5.59	10.01 ± 6.63	11.72 ± 7.94	10.36 ± 6.42
	B18	Phenylethyl acetate	3.21 ± 1.24	4.83 ± 2.67	5.60 ± 0.04	6.12 ± 0.19	6.22 ± 0.32	7.35 ± 1.49
	B19	Ethyl cetane	1.04 ± 0.25	1.56 ± 0.47	1.21 ± 0.84	2.06 ± 0.05	2.81 ± 2.23	2.12 ± 0.26
	B20	Octadecarbonate	2.00 ± 0.05	1.84 ± 0.23	1.24 ± 0.05	0.83 ± 0.25	0.75 ± 0.13	0.62 ± 0.41
	B21	Diethyl succinate	ND	ND	ND	ND	ND	ND
	B22	Isoamyl acetate	ND	ND	ND	ND	ND	ND
	B23	Ethyl myristate	ND	ND	ND	ND	ND	ND
	B24	Acetic acid−2‐phenylethyl ester	ND	ND	ND	ND	ND	ND
	B25	Ethyl decanoate	ND	ND	ND	ND	ND	ND
	B26	Ethyl undecanoate	ND	ND	ND	ND	ND	ND
	Total esters		83.29 ± 32.31	112.66 ± 44.21	121.22 ± 50.43	146.71 ± 52.97	160.36 ± 64.39	154.62 ± 54.79
Aldehyde	C1	Benzaldehyde	2.65 ± 1.39	2.12 ± 1.53	2.09 ± 1.32	1.23 ± 1.13	1.60 ± 0.19	1.54 ± 1.31
	C2	Phenylacetaldehyde	2.30 ± 0.85	2.24 ± 0.23	1.33 ± 0.68	1.46 ± 0.85	1.24 ± 1.07	1.15 ± 1.05
	C3	Furfural	2.13 ± 0.73	2.79 ± 0.35	2.93 ± 0.93	1.29 ± 0.21	1.10 ± 0.85	1.45 ± 1.25
	C4	Nonanal	2.06 ± 0.39	1.47 ± 0.81	0.20 ± 0.27	ND	ND	ND
	C5	Decanal	0.85 ± 0.25	0.53 ± 0.36	0.36 ± 0.18	ND	ND	ND
	C6	2‐Phenyl−2‐butyraldehyde	0.37 ± 0.33	0.38 ± 0.25	0.22 ± 0.13	ND	ND	ND
	C7	5‐Methylfurfural	0.97 ± 0.53	0.34 ± 0.19	0.73 ± 0.83	ND	ND	ND
	C8	3‐Methylbutyraldehyde	ND	ND	ND	ND	ND	ND
	C9	P‐methylbenzaldehyde	ND	ND	ND	ND	ND	ND
	Total aldehydes		11.33 ± 4.54	9.86 ± 3.75	7.85 ± 4.39	3.97 ± 2.18	3.94 ± 2.13	4.14 ± 3.83
Volatile acids	D1	Acetic acid	10.14 ± 0.53	7.97 ± 3.82	8.40 ± 2.28	9.73 ± 2.54	8.21 ± 4.24	7.19 ± 3.62
	D2	Hexanoic acid	2.15 ± 1.06	3.04 ± 0.23	2.40 ± 1.07	2.16 ± 0.25	1.47 ± 0.05	2.14 ± 0.25
	D3	Valeric acid	7.55 ± 3.27	7.27 ± 2.9	7.58 ± 3.69	7.30 ± 2.03	6.80 ± 1.43	6.20 ± 2.13
	D4	Caprylic acid	0.76 ± 0.14	0.81 ± 0.12	0.84 ± 0.06	0.80 ± 0.09	0.77 ± 0.18	0.63 ± 0.03
	D5	Butyrate	ND	ND	ND	ND	ND	ND
	D6	Decanoic acid	ND	ND	ND	ND	ND	ND
	D7	3‐Methylbutanoic acid	ND	ND	ND	ND	ND	ND
	Total acid compounds		20.60 ± 4.99	19.09 ± 7.15	19.22 ± 7.09	19.99 ± 4.95	17.25 ± 5.89	16.16 ± 6.07
Total volatile flavor compounds			606.09 ± 117.46	660.87 ± 126.57	624.19 ± 130.88	628.73 ± 130.87	614.45 ± 144.71	622.23 ± 136.62

Note

The results are expressed as means ± standard deviation with three replicates for Huangjiu sample during different brewing stages.

Abbreviation: ND, not detected.

During the 2, 4, and 6 days of the primary fermentation, 60 volatile flavor compounds were identified in the Huangjiu made from wheat Qu by hand and the Huangjiu made from wheat Qu by mechanical method contained 46. The main difference between the Huangjiu made from wheat Qu by hand and in the Huangjiu made from wheat Qu by mechanical method was in the numbers of alcohols and esters. Within 10, 20, and 30 days of the postfermentation, the Huangjiu brewed by handmade wheat Qu contained 58 kinds of volatile components. Within 10, 14, and 18 days of the postfermentation, the Huangjiu brewed by mechanical method wheat Qu contained 42 kinds of volatile components. Again, the two wines differed in the numbers of alcohols and esters. After a long fermentation, the Huangjiu made from wheat Qu by hand contained more abundant flavor ingredients, especially esters, than the Huangjiu made from wheat Qu by mechanical method. The results indicate that different Qu‐making methods had important effects on the content of components, particularly alcohol and esters, during the fermentation process of Huangjiu.

### 3.3 Changes in the main volatile aroma compounds during different brewing stages

**FIGURE 2:**
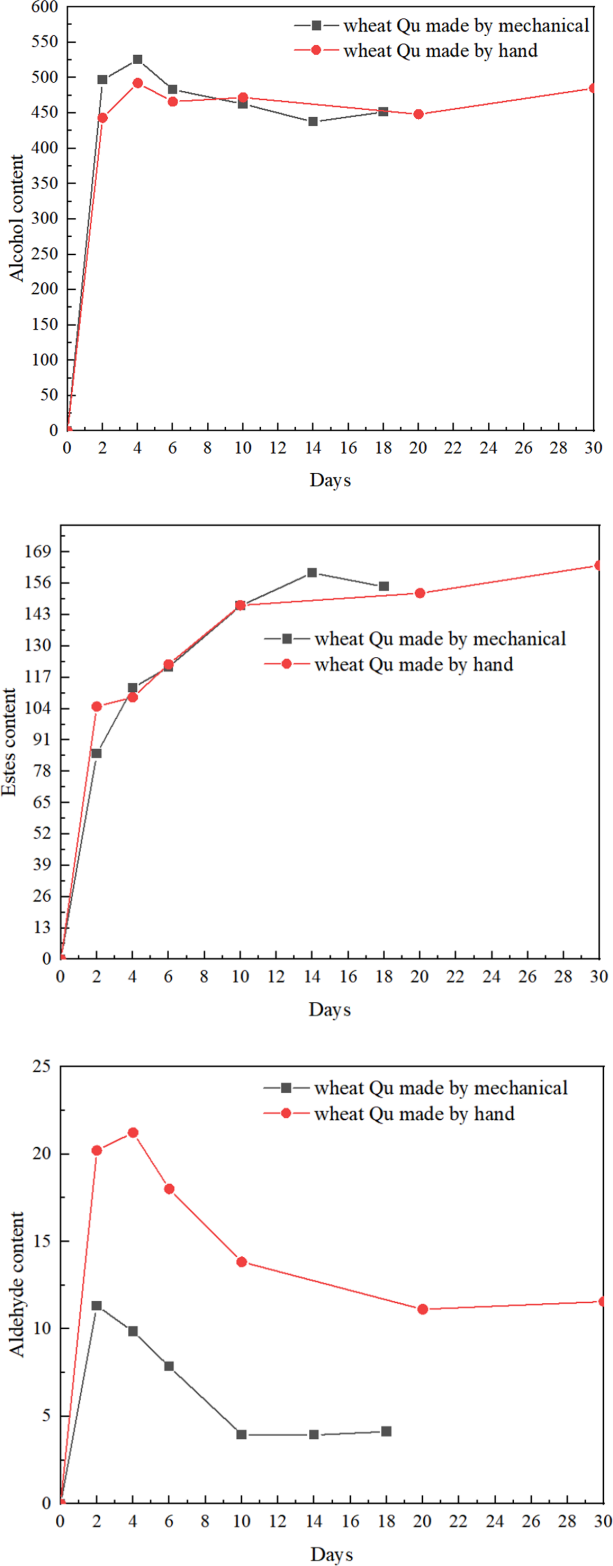
Changes in the main volatile flavor components of Huangjiu brewed from different wheat Qu during the fermentation process

The flavor of Huangjiu does not exist alone, but is formed by the interaction of alcohols, esters, and aldehydes (Cao et al., 2012; Xu et al., 2015). In terms of the types of volatile compounds, Huangjiu brewed from different wheat Qu mainly differed in the numbers of alcohols and esters. Furthermore, the amounts of other volatile flavor compounds were also obviously different. The changes in the main volatile aroma components (alcohols, esters, and aldehydes) during the brewing process are shown in Tables 2 and 3 and Figure 2.

When the Huangjiu began to be fermented, the microorganism grew rapidly, which led to the content of alcohols, esters, and aldehydes in the two kinds of Huangjiu increasing rapidly. After 2 days of prefermentation, the content of alcohols in Huangjiu increased slightly faster than that of esters and aldehydes. After 4 days of prefermentation, the content of alcohols and aldehydes in Huangjiu began to decrease, while the content of esters still showed an increasing trend. At the second time point of postfermentation, the alcohol content of two kinds of Huangjiu began to increase. This may have resulted from the azeotrope between the higher alcohols and water that would contain 15%–30% moisture. This is consistent with the results of Yang et al. (2017).

The key substances detected in alcohols include ethanol, isobutanol, β‐phenylethanol, and isopentanol, which account for much of the total content of alcohols. At the third time point of postfermentation, the content of ethanol in Huangjiu brewed from wheat Qu made by hand was lower than that of Huangjiu brewed from wheat Qu made by mechanical, but the difference was not significant. The content of isobutanol in the Huangjiu brewed from wheat Qu made by hand is higher than that of the Huangjiu brewed from wheat Qu made by mechanical. The content of isopentanol in the Huangjiu brewed from wheat Qu made by hand is lower than that in the Huangjiu brewed from wheat Qu made by mechanical. The content of β‐phenylethanol in the Huangjiu brewed from wheat Qu made by hand is lower than that in the Huangjiu brewed from wheat Qu made by mechanical. The total amount of alcohols in the Huangjiu brewed from wheat Qu made by hand is significantly higher than that in the Huangjiu brewed from wheat Qu made by mechanical. This may be because the content of amino acids in wheat Qu made by hand is higher than that of wheat Qu made by mechanical. During the brewing process of Huangjiu, the amino acids contained in Huangjiu are successively absorbed by yeast and then converted into higher alcohols by the Ehrlich pathway (Hazelwood et al., 2008).

Esters are an important component of flavoring compounds in Huangjiu, and most of them have the special floral and fruity aroma. Most of the ester substances in Huangjiu are ethyl ester (Tredoux et al., 2008). At the first‐time point of prefermentation, the ester content in the Huangjiu brewed from wheat Qu made by hand and the Huangjiu brewed from wheat Qu made by mechanical rose rapidly. After 6 days of prefermentation, the ester content in the Huangjiu brewed from wheat Qu made by hand was close to that of Huangjiu brewed from wheat Qu made by mechanical. At the second time point of postfermentation, the ester content in the Huangjiu brewed from wheat Qu made by hand was lower than that in the Huangjiu brewed from wheat Qu made by mechanical. However, at the third time point of postfermentation, the ester content in the Huangjiu brewed from wheat Qu made by hand was higher than that in the Huangjiu brewed from wheat Qu made by mechanical. This indicated that the wheat Qu made by hand had a significantly higher capacity to generate esters than the wheat Qu made by mechanical.

Aldehydes are produced in the late stage of Huangjiu fermentation and have special aroma, which can make the aroma of Huangjiu more intense and more harmonious. Acids play an important role in the coordination and balance of Huangjiu, which can increase the mellow feeling and reduce the bitterness and miscellaneous taste. The content of aldehydes and acids in Huangjiu is very low, but they also contribute to the flavor of Huangjiu. At the first‐time point of prefermentation, the content of aldehydes in the Huangjiu brewed from wheat Qu made by hand and the Huangjiu brewed from wheat Qu made by mechanical rose rapidly respectively. After 4 days of prefermentation, the content of aldehyde in Huangjiu brewed from wheat Qu made by hand and the Chinese Huangjiu brewed from wheat Qu made by mechanical began to decrease respectively. Until the last time point of postfermentation, the aldehyde content in the two Huangjiu was increased respectively. Aldehydes are produced by the oxidation of fatty acids and alcohols. The change of aldehydes reveals the important role of oxidation and chemical transformation in the formation of aldehydes.

### 3.4 PCA (principal component analysis) of volatile flavor compounds during different brewing stages

**FIGURE 3:**
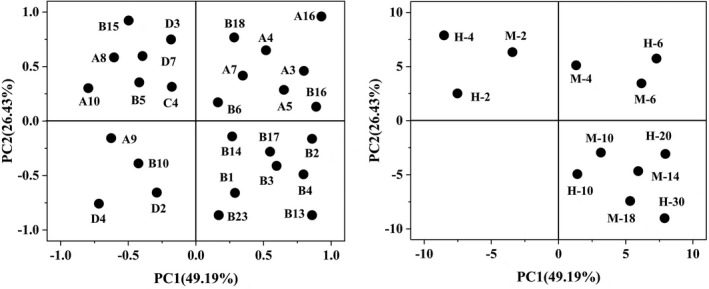
Biplot for principal component analysis (PCA) loadings for volatile flavor compounds and scores for the brewing stages of two Huangjiu. The codes of volatile flavor compounds and samples are defined in Tables 2 and 3. (H, the Huangjiu made from wheat Qu by hand; *M*, the Huangjiu made from wheat Qu by mechanical method)

Principal component analysis (PCA) (Bugli & Lambert, 2010; Fearn, 2008) was carried out to analyze the correlation between the volatile flavor compounds and the different brewing stages of two Huangjiu (Figure 3). Origin 2019b was used for PCA of volatile flavor substances. According to the principle of eliminating the variable of the corresponding maximum eigenvector of the principal component with the minimum eigenvalue, one variable is eliminated at a time, and then PCA is carried out with the remaining variables. The PCA results shown in Figure 3 reveal the close relationship between volatile flavor compounds, whereby the first and second principal compounds (PCs) explained the variance of 49.19% and 26.43%, respectively. As shown in Figure 3, the sixth day of prefermentation for Huangjiu brewed from wheat Qu made by hand, and the fourth day and sixth day of prefermentation for Huangjiu brewed from wheat Qu made by mechanical are all in the first quadrant. The main volatile flavor compounds are isopentanol, β‐ phenylethanol, n‐octanol, heptadiene glycol, propyl heptanoate, phenethyl formate, phenethyl acetate, and 2, 3‐butanediol. The second day of prefermentation and the fourth day of prefermentation for Huangjiu brewed from wheat Qu made by hand, and the second day of prefermentation of Huangjiu brewed from wheat Qu made by mechanical are all in the second quadrant. The main volatile flavor compounds are nonyl alcohol, propanol, ethyl valerate, ethyl benzoate, nonanal, and valeric acid. The 10th day of postfermentation, the 20th day of postfermentation, and the 30th day of postfermentation for Huangjiu brewed from wheat Qu made by hand are all in the fourth quadrant. The 10th day of postfermentation, the 14th day of postfermentation, and the 18th day of postfermentation for Huangjiu brewed from wheat Qu made by mechanical are all in the fourth quadrant. The main volatile flavor compounds are ethyl acetate, ethyl lactate, ethyl butyrate, ethyl propionate, isoamyl lactate, monoethyl succinate, and ethyl phenylacetate. The samples of Huangjiu brewed from wheat Qu made by hand and wheat Qu made by mechanical in the prefermentation stage and the postfermentation stage were very different. The prefermentation period and postfermentation period of Huangjiu samples are located in the upper part and lower part of Figure 3, respectively, which could well distinguish the volatile flavor substances in the prefermentation and postfermentation processes of Huangjiu samples.

### 3.5 Flavor assay based on the ratio of higher alcohols to esters

**FIGURE 4:**
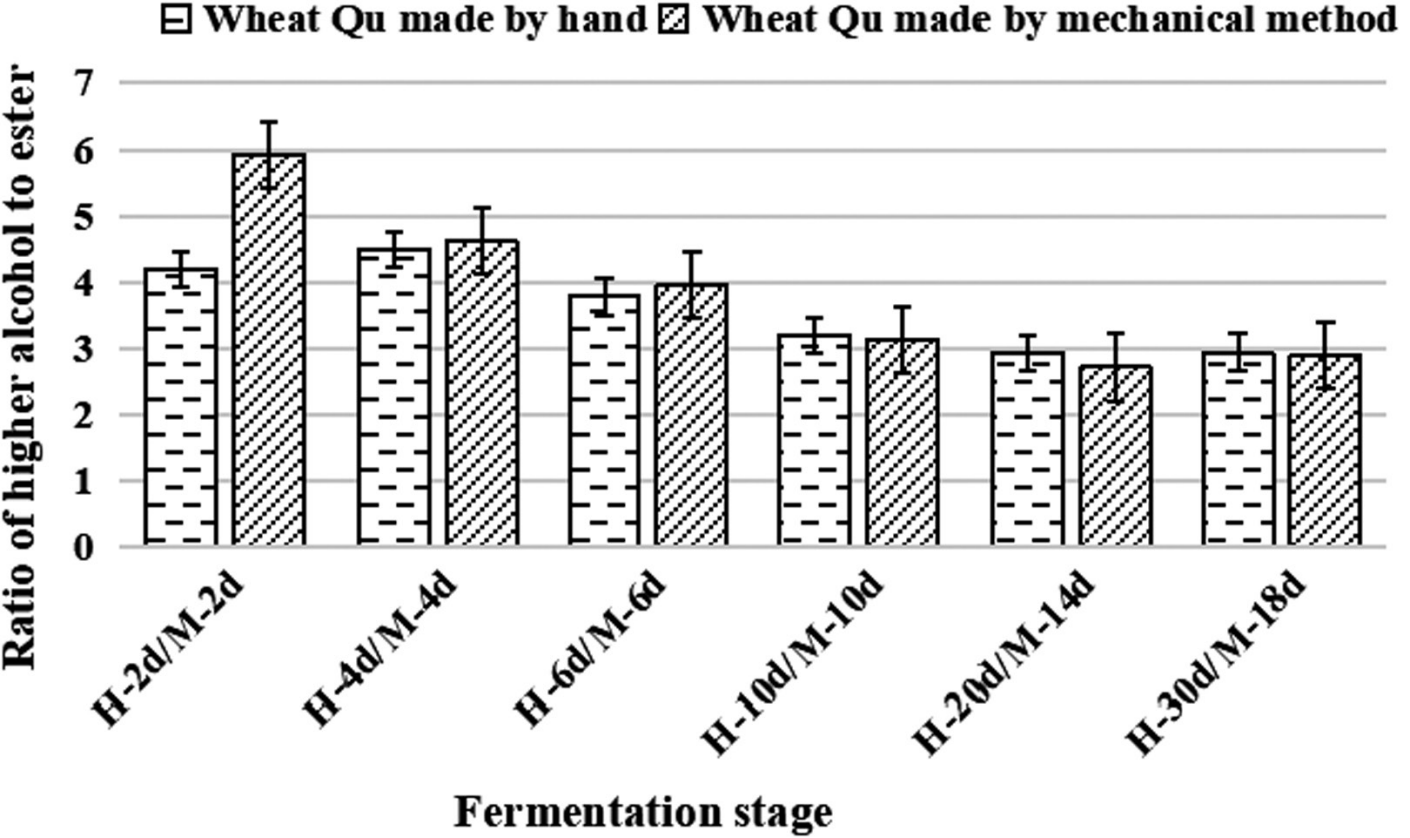
The ratio of higher alcohols to esters of Huangjiu fermented with different Qu‐making methods during the six brewing stages. (H, the Huangjiu made from wheat Qu by hand; *M*, the Huangjiu made from wheat Qu by mechanical method)

The aroma of Huangjiu is composed of many kinds of flavor compounds, among which the main aroma compounds are alcohols, esters, and aldehydes (Zhang et al., 2013). Advanced alcohol‐to‐ester ratio, which is a reliable index for evaluating the flavor characteristics of distillate spirits (Landaud et al., 2012; Procopio et al., 2011), was used to assess flavor in the Huangjiu brewed in this research. The fragrance of esters was more highlighted when the proportion was 2 and was thought to be moderate at a proportion between 3 and 4. If the ratio exceeded 5, the alcohol aroma would be highlighted. Advanced alcohol‐to‐ester ratio of the two Huangjiu (Figure 4) remained almost stable during the stages of postfermentation stage but apparently fluctuated by primary fermentation stage, ranging from 6 to 4. In the fermentation process, advanced alcohol‐to‐ester ratio of the two kinds of wine was kept at about 3, and the aroma of Huangjiu made by hand is superior to that made by mechanical method.

### 3.6 Heat map of wheat Qu and volatile flavor compounds produced by different processing methods

**FIGURE 5:**
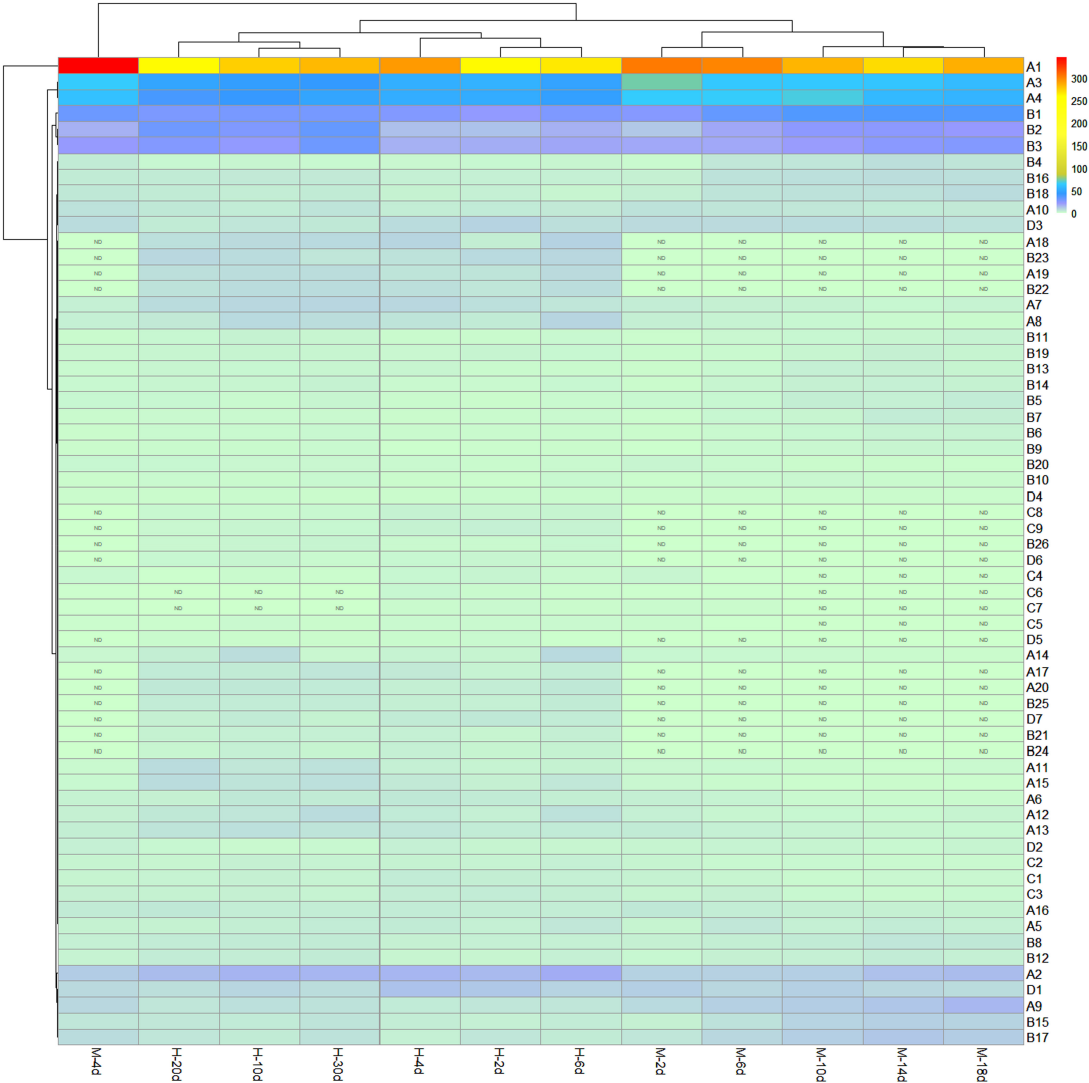
Heatmap study on Hungjiu and its main flavor compounds produced by wheat Qu produced by different Qu‐making methods. (H, The Huangjiu made from wheat Qu by hand; *M*, the Huangjiu made from wheat Qu by mechanical method; ND, not detected.)

From the volatile flavor compounds in Huangjiu and the heat map of wheat Qu obtained by different Qu‐making methods (Figure 5), in the brewing process of Huangjiu, the wheat Qu made by hand has an obvious effect on the production of 60 main flavor compounds, the wheat Qu made by mechanical method has an obvious effect on the production of 46 main flavor compounds, and there are obvious differences in the strength of their influence. The content and types of volatile flavor compounds in wheat QuHuangjiu brewed by hand were more than those made by mechanical method. Therefore, the difference of Huangjiu flavor produced by different Qu‐making methods may be caused by the metabolic differences of these substances.

**FIGURE 6:**
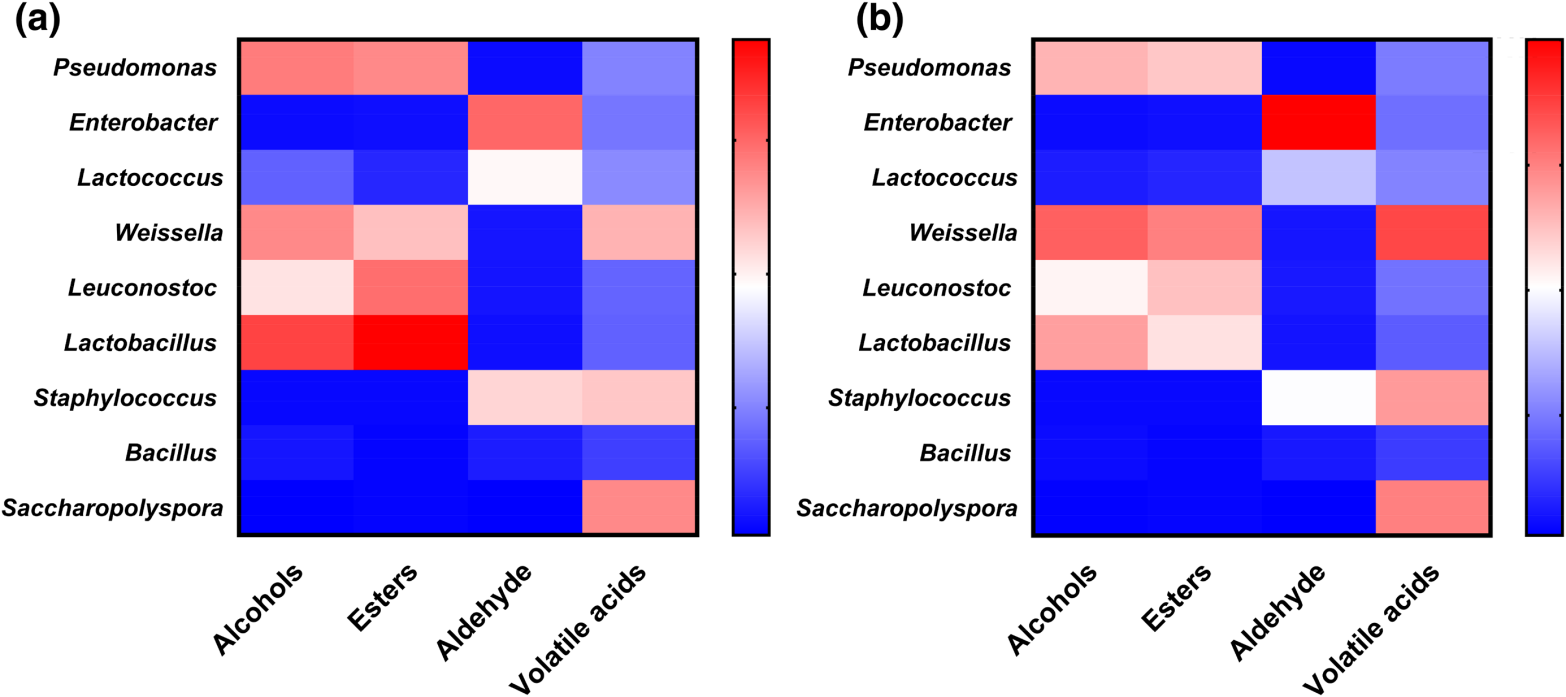
Relationship between flavor substances and bacterial composition. (a: The Huangjiu made from wheat Qu by hand; b: the Huangjiu made from wheat Qu by mechanical method.)

Alcohols and esters are the main flavor substances of Huangjiu (Zhou et al., 2019). As can be seen from the figure (Figure 6), *Pseudomonas*, *Weissella*, *Leuconostoc*, and *Lactobacillus* contribute significantly to alcohols and esters in Huangjiu. Compared with the mash of Huangjiu with mechanical Wheat Qu, the correlation between microorganisms and flavor substances is stronger in the mash of Huangjiu with manual Wheat Qu, which may be the diversity of microorganisms is better preserved in the mash of Huangjiu with manual Wheat Qu, so the flavor of Huangjiu with manual Wheat Qu is better than that with mechanical Wheat Qu.

### 3.7 Sensory evaluation

**FIGURE 7:**
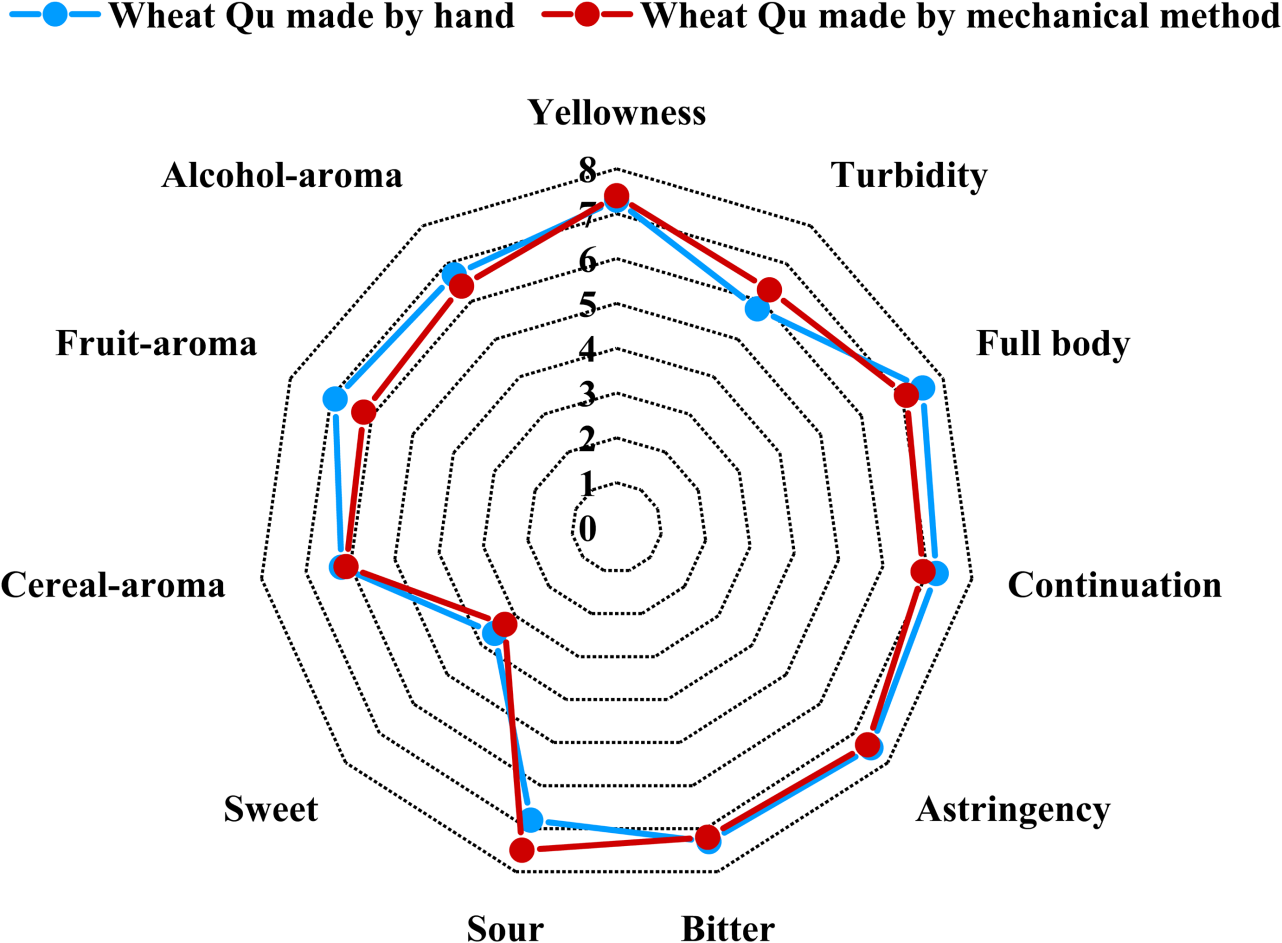
Sensory profiles of organoleptic attributes of Huangjiu made with different ways to make wheat Qu

Sensory analysis of the Huangjiu made by the different ways to make wheat Qu was performed by evaluating the organoleptic quality. The intensity ratings of the Huangjiu made from wheat Qu by hand and the Huangjiu made from wheat Qu by mechanical method are presented in (Figure 7). As for appearance, the Huangjiu made from wheat Qu by hand exhibited a lower turbidity score (5.7) among the two Huangjiu, while the two wines exhibited a similarly strong intensity rating in yellowness. The sweet flavor in the two Huangjiu seemed to have a lower descriptor score (<4.0) than the other aroma and taste. The two wines had big different ordinary values of fruit‐aroma attribute, which may have resulted from the different content esters produced through fermentation (Erten et al., 2007). Compared with the Huangjiu made from wheat Qu by mechanical method, the Huangjiu made from wheat Qu by hand has a little strong alcoholic aroma. The attribute levels of bitter flavor, astringency, and cereal‐aroma were generally similar between two wines, even though the Huangjiu made from wheat Qu by mechanical method showed a higher intensity of sour flavor. The Huangjiu brewed from wheat Qu by hand has been highly assessed in continuation and full‐body mouthfeel.

## 4 CONCLUSION

The volatile flavor compounds and chemical analysis of Huangjiu made by the wheat Qu made by hand and the wheat Qu made by mechanical method were investigated. Headspace–solid phase microextraction coupled with gas chromatography–mass spectrometry showed that wine brewed by different methods of making wheat Qu differed mainly in the numbers and amounts of alcohols and esters. In general, with the promotion of a fermentation process, the volatile components of handmade wheat Qu Huangjiu were always taller than that of the mechanical production of Huangjiu, which may be due to the differences in production technology between hand and mechanical wheat Qu. Wheat Qu made by mechanical method uses machine pressing, while wheat Qu made by manual method uses manual trampling. The pressure of the machine is often greater than the pressure of manual trampling, so the gap of wheat Qu made by mechanical method is smaller, the air content is less, and mold and yeast will not grow well. Moreover, the moisture content of mechanical wheat Qu is lower than that of manual wheat Qu, which also affects the growth and reproduction of microorganisms in the Qu to a certain extent. Therefore, the difference in composition of Huangjiu with different Qu‐making methods may be mainly on account of the difference in microorganism species in wheat Qu. The effect of microorganisms on the volatile flavor compounds of Huangjiu needs further study.

## Data Availability

The data used to support the findings of this study are available from the corresponding author, upon request.
